# Prevalence, determinants, and reasons for formula feeding among Palestinian mothers in the West Bank: a cross-sectional study

**DOI:** 10.3389/fpubh.2026.1908532

**Published:** 2026-09-03

**Authors:** Mohanad Jaber, Obada A. Bali, Mohammed Tahayneh, Ahmad Alhashlamon, Basel Abu Turkey, Mohammad Walid Radwan, Mostafa Ibraheem Abo Alrob, Ali Hamad, Nidal B. M. Nazzal, Ibraheem AbuAlrub

**Affiliations:** 1 Clinical Medicine Department, Faculty of Medicine, Palestine Polytechnic University, Hebron, Palestine; 2 Department of Clinical Medical Sciences, Faculty of Medicine and Health Sciences, Palestine Polytechnic University, Hebron, Palestine; 3 Basic Medicine Department, Faculty of Medicine, Palestine Polytechnic University, Hebron, Palestine; 4 College of Health and Agricultural Sciences, University College Dublin, Dublin, Ireland; 5 Jenin Governmental Hospital, Jenin, Palestine; 6 Department of Clinical Medical Sciences, Faculty of Medicine, Palestine Polytechnic University, Hebron, Palestine

**Keywords:** antenatal care, breastfeeding, cross-sectional study, exclusive breastfeeding, formula feeding, infant feeding, Palestinian mothers, West Bank

## Abstract

**Background:**

Exclusive breastfeeding rates in Palestine remain below WHO targets, and formula use is prevalent among infants in the first two years of life. Evidence on the specific factors associated with formula introduction in the Palestinian context is limited.

**Objectives:**

To assess the prevalence of formula feeding among Palestinian mothers of infants aged 0–24 months, identify the primary reasons for formula introduction, and determine the independent sociodemographic, maternal, infant, and perinatal predictors of formula use.

**Methods:**

A community-based cross-sectional study was conducted between October 2024 and January 2025 in three primary healthcare centers across the northern, central, and southern West Bank (Nablus, Ramallah, and Hebron governorates). A total of 506 mothers meeting eligibility criteria were enrolled using consecutive sampling. Data were collected via a structured, investigator-developed Arabic questionnaire covering maternal sociodemographic characteristics, chronic disease history, infant feeding practices, and perinatal variables. Bivariable analyses (chi-square and Fisher’s exact tests) and multivariable binary logistic regression were used to identify factors independently associated with formula feeding.

**Results:**

Mixed feeding was the most common feeding practice (62.3%), followed by exclusive breastfeeding (29.1%) and exclusive formula feeding (8.7%). Overall, 70.9% of mothers introduced formula within the first 24 months, primarily due to perceived low milk supply (32.3%) and time constraints (25.9%). Independent predictors of formula feeding included antenatal care attendance (aOR = 5.34, 95% CI: 1.45–19.70) and having ≥3 children (aOR = 1.88, 95% CI: 1.04–3.42), while breastfeeding education (aOR = 0.59, 95% CI: 0.35–0.98) and breastfeeding initiation within 24 h of birth (aOR = 0.27, 95% CI: 0.09–0.85) were protective factors.

**Conclusion:**

Formula feeding is highly prevalent among Palestinian mothers, with mixed feeding being the dominant practice. Antenatal breastfeeding education and targeted postnatal support for multiparous mothers are priority areas for intervention within Palestinian maternal health services. The counterintuitive positive association between antenatal care attendance and formula feeding underscores the need to audit and strengthen breastfeeding-promotional content within antenatal consultations.

## 1 Introduction

Breastfeeding is the optimal method of infant feeding and is recommended by the World Health Organization (WHO) and UNICEF as exclusive nutrition for the first six months of life, followed by continued breastfeeding alongside complementary foods until at least two years of age (1). Breastfeeding provides substantial health benefits for both infants and mothers, including protection against infectious diseases, improved long-term health outcomes, and reduced maternal risks of breast cancer and other chronic conditions (2).

Despite these benefits, exclusive breastfeeding rates remain below recommended targets worldwide. At the same time, infant formula use has increased in many regions, including the Middle East and North Africa (3, 4). Formula feeding has been associated with higher risks of infections for the offspring and may contribute to early cessation of breastfeeding (5). Common reasons for formula introduction include perceived insufficient milk supply, maternal employment, lack of breastfeeding support, and exposure to formula marketing (6–8).

In the Eastern Mediterranean region, breastfeeding initiation is generally high, but exclusive breastfeeding declines rapidly during the first months of life (6). Similar patterns have been reported in Palestine, where most mothers initiate breastfeeding, yet exclusive breastfeeding rates during the first six months remain relatively low (7, 8). Factors such as limited lactation support, healthcare system challenges, and socioeconomic pressures may contribute to this trend (9, 10).

Although several Palestinian studies have examined breastfeeding practices revealing that 43.8% of Palestinian infants are exclusively breast fed, evidence specifically addressing formula feeding and its determinants remains limited. Understanding the factors associated with formula use is essential for developing effective interventions to support optimal infant feeding.

Therefore, this study aimed to assess the prevalence of formula feeding among mothers of infants aged 0–24 months in the West Bank, Palestine, identify the reasons for formula use, and determine the sociodemographic, maternal, infant, and perinatal factors associated with formula feeding.

## 2 Methods

### 2.1 Study design and setting

This study employed a community-based, cross-sectional design to examine the prevalence of formula feeding and its associated factors among Palestinian mothers. Cross-sectional studies are appropriate for estimating the prevalence of health-related behaviors and identifying sociodemographic and clinical correlates at a single time point, in accordance with the STROBE (Strengthening the Reporting of Observational Studies in Epidemiology) reporting guidelines for observational research (11). Data were collected between 1 October 2024 and 25 January 2025.

The study was conducted in the West Bank, Palestine. Three primary healthcare centres (PHCs) operated by the Palestinian Ministry of Health were purposively selected to represent the three geographic sub-regions of the West Bank: one centre in the northern region (Nablus governorate), one in the central region (Ramallah governorate), and one in the southern region (Hebron governorate). This multi-site approach was adopted to improve the geographic representativeness of the sample across diverse urban and semi-urban communities.

### 2.2 Participants

#### 2.2.1 Eligibility criteria

The target population comprised all mothers aged 18 to 45 years who had at least one living infant aged 0 to 24 months and who were residing in the West Bank, Palestine at the time of data collection. Mothers attending the selected PHCs for routine infant vaccination or well-child visits were approached for enrolment.

Mothers were eligible if they: (i) were aged between 18 and 45 years; (ii) had an infant aged 0 to 24 months at the time of the interview; (iii) were residing in the catchment area of one of the three selected PHCs; and (iv) provided written informed consent to participate.

Mothers were excluded if they: (i) were critically ill or otherwise medically unable to respond (defined operationally as requiring active inpatient medical treatment, including unconsciousness, severe acute illness, or psychiatric crisis precluding informed consent) or (ii) had introduced formula feeding for the first time after the infant’s first birthday (excluded because the primary outcome was formula use within the infant feeding period for children under 12 months), and including late-starting formula users would have introduced heterogeneous feeding-pattern types inconsistent with the study’s focus.

### 2.3 Sample size determination

The required minimum sample size was calculated using Cochran’s sample size formula for estimating a single population proportion: *n* = Z^2^P(1 − P)/d^2^, where Z = 1.96, *p* = 0.50, and d = 0.05 and a population of 1 million female in the reproductive age. Applying this formula yielded a minimum sample size of 385.

### 2.4 Sampling procedure

A convenience sampling approach was employed in this study. All eligible mothers attending to three selected centers during the data collection period were invited to participate until the target number of participants per site was reached. The centers were selected each in different area in the west bank in a center with access to different patient from city, villages and town to insure geographical representativeness.

### 2.5 Data collection

#### 2.5.1 Instrument

Data were collected using a structured, investigator-developed questionnaire administered in Arabic. The questionnaire was developed *de novo* by the research team supervised by the principal investigator, with items adopted by previously published instruments used in comparable Palestinian and Eastern Mediterranean infant feeding studies (12, 13). The instrument was translated into English and back-translated into Arabic by 2 native English speakers to ensure semantic equivalence. Content was validated by 2 independent reviewers to insure validity and suitability of instrument items to the research topic. The questionnaire covered six domains: (i) maternal sociodemographic characteristics; (ii) maternal chronic disease history; (iii) infant characteristics and feeding practices; (iv) antenatal and perinatal history; (v) timing and reasons for formula introduction; and (vi) receipt of breastfeeding-related education or counseling. The full study instrument can be seen in Supplementary material 1.

#### 2.5.2 Pilot testing and data collector training

The questionnaire was piloted on 51 mothers (approximately 10% of the target sample) from a PHC not included in the main study. Minor changes (some explanations and grammar corrections) four trained data collectors were recruited and provided with a standardized one-day training session.

#### 2.5.3 Data collection procedure

Questionnaires were administered face-to-face by trained data collectors in the waiting areas or consultation rooms of the PHCs. After the study purpose was explained and written consent obtained, the data collector read each question aloud in standard Arabic and recorded the mother’s response.

### 2.6 Study variables

The primary outcome variable was formula feeding, defined as the introduction of any commercial infant formula milk at any point during the first 12 months of infant life, regardless of whether formula was used as a supplement to or a substitute for breastfeeding. This was operationalized as a binary variable (yes/no). Type of milk Feeding was defined as the type of milk feeding that have been introduced to the infant before the age of 1 year outcome was (Exclusive breastfeed, Exclusive formula feed, formula and breast feed). Explanatory variables were organized into four domains: (i) maternal sociodemographic factors; (ii) maternal chronic disease history; (iii) infant characteristics; and (iv) antenatal and perinatal factors. Supplementary material 2 have all study variables with their operational definitions.

### 2.7 Bias

Selection bias was mitigated by recruiting from three PHCs across geographically distinct regions. Information bias was addressed through standardized questionnaire administration and back-translation procedures. Recall bias is an inherent limitation for mothers of older infants. Social desirability bias may have led to over-reporting of exclusive breastfeeding; data collectors were trained to administer questions in a neutral manner. Residual confounding from unmeasured variables cannot be entirely excluded.

### 2.8 Statistical analysis

All data were entered and analyzed in IBM SPSS Statistics (version 26.0). Descriptive statistics were computed for all variables. Bivariable analyses used the chi-square test and crude odds ratios with 95% CIs were calculated. Variables significantly associated with formula feeding in bivariable analysis (*p* < 0.05), together with clinically relevant covariates, were entered into a multivariable binary logistic regression model (Enter method). Model fit was assessed using the Hosmer–Lemeshow test and Nagelkerke R^2^. Statistical significance was set at *p* < 0.05 (two-sided).

### 2.9 Ethical considerations

The study was conducted in accordance with the principles of the Declaration of Helsinki. Ethical approval was obtained from the Institutional Review Board of Palestine Polytechnic University (Reference No.: EA/2025/81). Permission was granted by the Palestinian Ministry of Health. Participation was entirely voluntary, and written informed consent was obtained from all participants. Data were anonymised at the point of collection.

## 3 Results

### 3.1 Response rate

**Figure 1:**
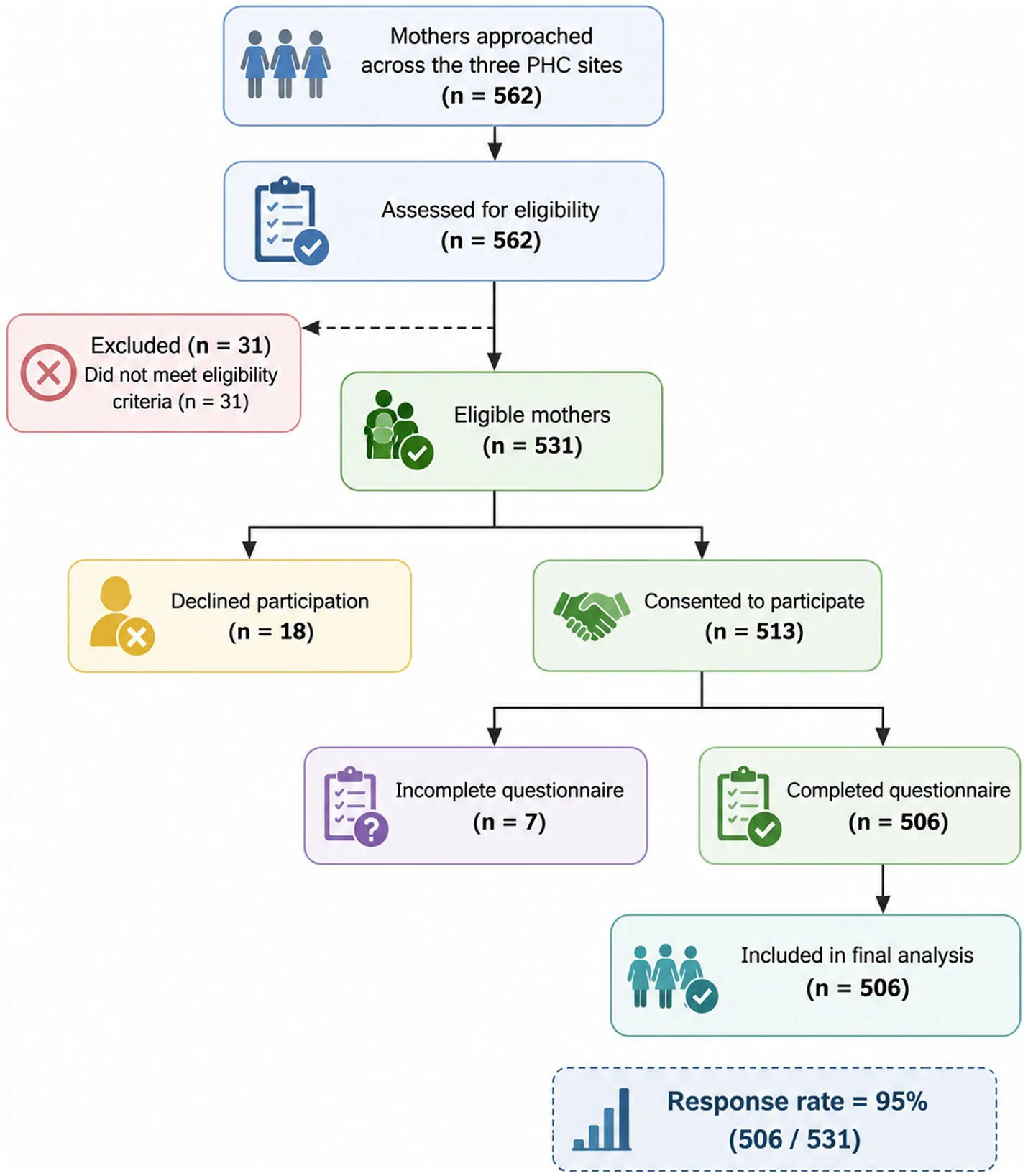
Participant flow diagram. Flow of mothers through eligibility assessment, participation, questionnaire completion, and inclusion in the final analysis.

A total of 562 mothers were approached across the three PHC sites; of these, 531 met the eligibility criteria. 18 mothers declined to participate, and seven did not complete the questionnaire yielding a response rate of 95% (506/531) (Figure 1).

### 3.2 Sociodemographic characteristics of study participants

The majority of participants (46.2%) were aged 25–34 years, followed by those under 25 years (28.3%) and those aged 35 years or older (25.5%). Nearly all participants were married (98.0%). With respect to educational attainment, approximately half of the sample (47.0%) held a bachelor’s degree. The most common employment status was housewife (58.9%). More than half of participants (54.7%) had three or more children, and 25.5% were primiparous. These characteristics are detailed in Table 1.

**Table 1:** Sociodemographic characteristics of study participants (*N* = 506).

Variable	Frequency (%)
Maternal age group	
Under 25 years	143 (28.3%)
25–34 years	234 (46.2%)
35 years and over	129 (25.5%)
Marital status	
Married	496 (98.0%)
Divorced	4 (0.8%)
Widowed	6 (1.2%)
Educational level	
Basic education	66 (13.0%)
Secondary school	103 (20.4%)
Diploma	75 (14.8%)
Bachelor’s degree	238 (47.0%)
Master’s or PhD	24 (4.7%)
Employment status	
Housewife	298 (58.9%)
Student	35 (6.9%)
Government employee	83 (16.4%)
Private employee	50 (9.9%)
Freelancer	23 (4.5%)
Health-related field	17 (3.4%)
Parity (number of children)	
1 (primiparous)	129 (25.5%)
2	100 (19.8%)
3 or more	277 (54.7%)
Family history of genetic disorders	
Yes	38 (7.5%)
No	468 (92.5%)
Maternal weight	
< 60 kg	134 (26.5%)
60–80 kg	307 (60.7%)
> 80 kg	65 (12.8%)

### 3.3 Prevalence of chronic diseases among participants

Chronic disease burden was relatively low. Breast disorders were the most frequently reported condition (6.3%), followed by thyroid disease (5.9%), malnutrition (4.2%), hypertension (3.2%), heart disease (1.8%), and diabetes mellitus (1.2%). Prior breast surgery was reported by only 0.4% of participants (Table 2).

**Table 2:** Prevalence of chronic conditions among study participants (*N* = 506).

Condition	Frequency (%)
Hypertension	16 (3.2%)
Diabetes mellitus	6 (1.2%)
Heart disease	9 (1.8%)
Breast disorder	32 (6.3%)
Thyroid disease	30 (5.9%)
Malnutrition	21 (4.2%)
Prior breast surgery	2 (0.4%)

### 3.4 Perinatal and antenatal characteristics

The majority of deliveries (73.3%) occurred via vaginal delivery. Delivery complications were reported in 12.8% of cases, and 13.8% of neonates required NICU admission. Most infants (81.0%) were born with a birthweight in the normal range. The vast majority of participants (93.7%) received antenatal care, and breastfeeding education was received by 75.5% of participants (Table 3).

**Table 3:** Perinatal and antenatal characteristics of study participants (*N* = 506).

Variable	Frequency (%)
Mode of delivery	
Vaginal delivery	371 (73.3%)
Cesarean section	135 (26.7%)
Delivery complications	
Yes	65 (12.8%)
No	441 (87.2%)
NICU admission	
Yes	70 (13.8%)
No	436 (86.2%)
Birth weight	
< 2,500 g (low birthweight)	77 (15.2%)
2,500–4,000 g (normal)	410 (81.0%)
> 4,000 g (macrosomia)	19 (3.8%)
Gestational age at delivery	
< 36 weeks (preterm)	60 (11.9%)
36–40 weeks (term)	382 (75.5%)
> 40 weeks (post-term)	64 (12.6%)
Antenatal care	
Received antenatal care	474 (93.7%)
< 3 visits	30 (5.9%)
≥ 3 visits	476 (94.1%)
Pregnancy complications	
Medical problems during pregnancy	100 (19.8%)
Hypertensive disorders of pregnancy	49 (9.7%)
Gestational diabetes mellitus	45 (8.9%)
Breastfeeding education	
Received antenatal breastfeeding education	382 (75.5%)

### 3.5 Infant characteristics and feeding practices

The majority of infants were male (55.3%). Mixed feeding (breastfeeding combined with formula) was the predominant mode, practiced by 62.3% of mothers. Exclusive breastfeeding was reported by 29.1% of participants, while exclusive formula feeding was the least common (8.7%). Among the 462 mothers who initiated breastfeeding, more than half (55.4%) did so within the first hour of birth. Among the 359 mothers who introduced formula, the largest proportion (27.0%) began within the first hour (Table 4).

**Table 4:** Infant characteristics and feeding practices (*N* = 506).

Variable	Frequency (%)
Infant age	
0–3 months	155 (30.6%)
3–6 months	70 (13.8%)
6–9 months	58 (11.5%)
9–12 months	39 (7.7%)
12–18 months	53 (10.5%)
18–24 months	131 (25.9%)
Infant sex	
Male	280 (55.3%)
Female	226 (44.7%)
Type of milk feeding	
Exclusive breastfeed	147 (29.1%)
Exclusive formula feed	44 (8.7%)
Formula and breast feed	315 (62.3%)
Timing of breastfeeding initiation (*n* = 462)	
Within 1 h	256 (55.4%)
After 1 h and before the end of the first 24 h	140 (30.3%)
1–3 days	32 (6.9%)
More than 3 days	34 (7.4%)
Timing of formula introduction (*n* = 359)	
Within 1 h	97 (27.0%)
After 1 h and before the end of the first 24 h	39 (10.9%)
1–3 days	41 (11.4%)
3 days – 1 month	70 (19.5%)
1–3 months	53 (14.8%)
3–6 months	41 (11.4%)
6–12 months	18 (5.0%)

Formula feeding by infant age group was as follows: among infants aged 0–6 months (*n* = 225), 58.7% (95% CI: 52.0–65.1%) had received formula at some point; among infants aged 6–12 months (*n* = 97), 75.3% (95% CI: 65.6–83.3%) had been given formula; and among infants aged 12–24 months (*n* = 184), 78.8% (95% CI: 72.4–84.3%) had been introduced to formula. The increase in formula use across age groups is consistent with progressive supplementation as exclusive breastfeeding declines over the first two years of life.

### 3.6 Feeding practices and reasons for formula introduction

Of the 506 participants, 91.3% (95% CI: 88.4–93.7%) reported breastfeeding their infant at some point, while 70.9% (95% CI: 66.7–74.8%) reported introducing formula milk. The most commonly cited reason was perceived low milk supply (32.3%), followed by maternal time constraints (25.9%), advice from healthcare providers (15.0%), other medical conditions (13.4%), infant refusal of the breast (8.1%), and advice from relatives (5.3%). These findings are presented in Table 5.

**Table 5:** Feeding practices and reasons for formula introduction.

Variable	Frequency (%)
Any breastfeeding (yes)	462 (91.3%)
Any formula feeding (yes)	359 (70.9%)
Primary reason for formula introduction (*n* = 359)	
Maternal factors	
Perceived low milk supply	116 (32.3%)
Maternal time constraints / busy schedule	93 (25.9%)
Advice from healthcare providers	54 (15.0%)
Other medical conditions (mother or infant)^†^	48 (13.4%)
Advice from relatives	19 (5.3%)
Infant factors	
Infant refusal of the breast	29 (8.1%)

^†^Includes cesarean section recovery, NICU admission, multiple gestation, infant intolerance to breast milk, and concerns about breast shape.

### 3.7 Factors associated with formula feeding: bivariable analysis

Bivariable analyses identified maternal educational level (*p* = 0.004), employment status (*p* < 0.001), and parity (*p* = 0.004) as significantly associated with formula feeding. Maternal malnutrition was the only chronic disease variable reaching significance (*p* = 0.044). Timing of breastfeeding initiation (*p* < 0.001), low birthweight (*p* = 0.003), NICU admission (*p* = 0.008), delivery complications (*p* = 0.044), and antenatal care attendance (*p* = 0.011) were significant perinatal predictors. Full bivariable results are presented in Table 6.

**Table 6:** Bivariable analysis of factors associated with formula feeding using chi square test.

Variable	Category	Formula: Yes *n* (%)	Formula: No *n* (%)	*p*-value
Sociodemographic variables				
Maternal age	< 25 years	108 (75.5%)	35 (24.5%)	0.330
	25–34 years	160 (68.4%)	74 (31.6%)	
	≥ 35 years	91 (70.5%)	38 (29.5%)	
Marital status	Married	353 (71.2%)	143 (28.8%)	0.632
	Divorced	2 (50.0%)	2 (50.0%)	
	Widowed	4 (66.7%)	2 (33.3%)	
Educational level	Basic education	46 (69.7%)	20 (30.3%)	0.004
	Secondary school	60 (58.3%)	43 (41.7%)	
	Diploma	49 (65.3%)	26 (34.7%)	
	Bachelor’s degree	185 (77.7%)	53 (22.3%)	
	Master’s / PhD	19 (79.2%)	5 (20.8%)	
Employment status	Housewife	189 (63.4%)	109 (36.6%)	< 0.001
	Student	29 (82.9%)	6 (17.1%)	
	Government employee	72 (86.7%)	11 (13.3%)	
	Private employee	42 (84.0%)	8 (16.0%)	
	Freelancer	14 (60.9%)	9 (39.1%)	
	Health-related field	13 (76.5%)	4 (23.5%)	
Parity	1 (primiparous)	103 (79.8%)	26 (20.2%)	0.004
	2 children	76 (76.0%)	24 (24.0%)	
	≥ 3 children	180 (65.0%)	97 (35.0%)	
Family history of genetic disorders	Yes	29 (76.3%)	9 (23.7%)	0.449
	No	330 (70.5%)	138 (29.5%)	
Maternal weight	< 60 kg	102 (76.1%)	32 (23.9%)	0.306
	60–80 kg	212 (69.1%)	95 (30.9%)	
	> 80 kg	45 (69.2%)	20 (30.8%)	
Chronic disease variables				
Hypertension	Yes	13 (81.3%)	3 (18.8%)	0.356
	No	346 (70.6%)	144 (29.4%)	
Diabetes mellitus	Yes	5 (83.3%)	1 (16.7%)	0.501
	No	354 (70.8%)	146 (29.2%)	
Heart disease	Yes	9 (100.0%)	0 (0.0%)	0.053
	No	350 (70.4%)	147 (29.6%)	
Breast disorder	Yes	21 (65.6%)	11 (34.4%)	0.493
	No	338 (71.3%)	136 (28.7%)	
Thyroid disease	Yes	21 (70.0%)	9 (30.0%)	0.906
	No	338 (71.0%)	138 (29.0%)	
Malnutrition	Yes	19 (90.5%)	2 (9.5%)	0.044
	No	340 (70.1%)	145 (29.9%)	
Infant characteristics				
Infant age	0–3 months	102 (65.8%)	53 (34.2%)	0.663
	3–6 months	50 (71.4%)	20 (28.6%)	
	6–9 months	42 (72.4%)	16 (27.6%)	
	9–12 months	29 (74.4%)	10 (25.6%)	
	12–18 months	38 (71.7%)	15 (28.3%)	
	18–24 months	98 (74.8%)	33 (25.2%)	
Infant sex	Male	197 (70.4%)	83 (29.6%)	0.744
	Female	162 (71.7%)	64 (28.3%)	
Timing of breastfeeding initiation (*n* = 462)	Within 1 h	154 (60.2%)	102 (39.8%)	< 0.001
	Within 1 day	104 (74.3%)	36 (25.7%)	
	1–3 days	27 (84.4%)	5 (15.6%)	
	> 3 days	30 (88.2%)	4 (11.8%)	
Perinatal and antenatal variables				
Mode of delivery	Vaginal delivery	257 (69.3%)	114 (30.7%)	0.169
	Cesarean section	102 (75.6%)	33 (24.4%)	
Delivery complications	Yes	53 (81.5%)	12 (18.5%)	0.044
	No	306 (69.4%)	135 (30.6%)	
NICU admission	Yes	59 (84.3%)	11 (15.7%)	0.008
	No	300 (68.8%)	136 (31.2%)	
Birth weight	< 2,500 g	67 (87.0%)	10 (13.0%)	0.003
	2,500–4,000 g	278 (67.8%)	132 (32.2%)	
	> 4,000 g	14 (73.7%)	5 (26.3%)	
Antenatal care	Yes	330 (69.6%)	144 (30.4%)	0.011
	No	29 (90.6%)	3 (9.4%)	
Antenatal visits	< 3	21 (70.0%)	9 (30.0%)	0.906
	≥ 3	338 (71.0%)	138 (29.0%)	
Medical problems in pregnancy	Yes	77 (77.0%)	23 (23.0%)	0.137
	No	282 (69.5%)	124 (30.5%)	
Breastfeeding education	Yes	279 (73.0%)	103 (27.0%)	0.069
	No	80 (64.5%)	44 (35.5%)	
Hypertensive disorders of pregnancy	Yes	38 (77.6%)	11 (22.4%)	0.284
	No	321 (70.2%)	136 (29.8%)	
Gestational diabetes	Yes	34 (75.6%)	11 (24.4%)	0.476
	No	325 (70.5%)	136 (29.5%)	
Gestational age	< 36 weeks	50 (83.3%)	10 (16.7%)	0.078
	36–40 weeks	264 (69.1%)	118 (30.9%)	
	> 40 weeks	45 (70.3%)	19 (29.7%)	

### 3.8 Independent predictors of formula feeding: multivariable logistic regression

After adjusting for potential confounders, four independent predictors of formula feeding were identified (Table 7). Antenatal care attendance was the strongest predictor (adjusted OR = 5.34, 95% CI: 1.45–19.70; *p* = 0.012). Having three or more children was independently associated with increased odds of formula feeding compared with primiparous mothers (adjusted OR = 1.88, 95% CI: 1.04–3.42; *p* = 0.037). Conversely, receipt of breastfeeding education was associated with reduced odds of formula feeding (adjusted OR = 0.59, 95% CI: 0.35–0.98; *p* = 0.043). Initiating breastfeeding within the first 24 h (but not within the first hour) was associated with significantly lower formula use (adjusted OR = 0.27, 95% CI: 0.09–0.85; *p* = 0.025).

**Table 7:** Multivariable logistic regression analysis of factors independently associated with formula feeding.

Variable	B Coefficient	Adjusted OR (95% CI)	*p*-value
Educational level (ref: basic education) overall *p* value			0.970
High school	0.312	1.37 (0.28–6.57)	0.698
Diploma	0.029	1.03 (0.23–4.56)	0.970
Bachelor’s degree	0.100	1.11 (0.25–4.88)	0.895
Master’s / PhD	0.100	1.11 (0.28–4.34)	0.886
Employment status (ref: housewife) overall *p* value			0.002
Student	−0.835	0.43 (0.09–2.01)	0.286
Government employee	0.264	1.30 (0.23–7.34)	0.764
Private employee	0.833	2.30 (0.46–11.54)	0.312
Freelancer	0.262	1.30 (0.28–6.08)	0.739
Health-related profession	−0.585	0.56 (0.10–3.02)	0.497
Number of children (ref: 1 child) overall *p* value			0.062
Two children	0.487	1.63 (0.90–2.94)	0.107
Three or more children	0.634	1.88 (1.04–3.42)	0.037*
Maternal heart disease	—	—	0.999
Maternal malnutrition	−0.687	0.50 (0.09–2.72)	0.425
Timing of breastfeeding initiation (ref: within 1 h) overall *p* value			0.002
Within 1 day	−1.317	0.27 (0.09–0.85)	0.025*
1–3 days	−0.576	0.56 (0.17–1.87)	0.348
> 3 days	0.051	1.05 (0.23–4.74)	0.947
Delivery complications	−0.404	0.67 (0.31–1.45)	0.307
NICU admission	−0.039	0.96 (0.40–2.29)	0.931
Birth weight (ref: 2,500–4,000 g) overall *p* value			0.143
< 2,500 g	0.781	2.18 (0.52–9.23)	0.288
> 4,000 g	−0.065	0.94 (0.28–3.18)	0.917
Antenatal care attendance	1.676	5.34 (1.45–19.70)	0.012*
Receipt of breastfeeding education	−0.537	0.59 (0.35–0.98)	0.043*
Gestational age (ref: 36–40 weeks) overall *p* value			0.749
< 36 weeks	0.152	1.16 (0.42–3.21)	0.769
> 40 weeks	−0.137	0.87 (0.45–1.69)	0.686

*Bold *p*-values indicate statistical significance (*p* < 0.05). OR = odds ratio; CI = confidence interval.

**Maternal heart disease have been excluded from the model due to sparce data.

## 4 Discussion

This cross-sectional study of 506 Palestinian mothers revealed that mixed feeding was the predominant infant feeding mode, practiced by 62.3% of participants, while exclusive breastfeeding was reported by only 29.1% and exclusive formula feeding by 8.7%. Multivariable logistic regression identified three independent predictors: antenatal care attendance (adjusted OR = 5.34), having three or more children (adjusted OR = 1.88), and receipt of breastfeeding education (adjusted OR = 0.59, protective). Initiating breastfeeding within the first 24 h was also independently associated with lower formula use (adjusted OR = 0.27).

The counterintuitive positive association between antenatal care attendance and formula feeding warrants careful consideration. Mothers who engaged with antenatal services may have received recommendations from healthcare providers to supplement with formula, particularly in the context of perceived or actual clinical indications. Moreover, greater health-system engagement may translate into higher awareness of commercial formula products. This finding does not imply that antenatal care is harmful to breastfeeding per se; rather, it highlights the possibility that breastfeeding-promotional content in antenatal consultations may be insufficiently robust.

The association between having three or more children and increased formula use is consistent with the logistical demands faced by multiparous mothers. Caring for multiple dependants simultaneously may constrain time for exclusive breastfeeding, reduce opportunities for on-demand nursing, or increase maternal fatigue, plausible pathways through which higher parity is linked to formula introduction.

Among self-reported reasons for formula introduction, perceived low milk supply (32.3%) and maternal time constraints (25.9%) were most frequently cited. Perceived insufficiency of milk is widely recognized as a leading driver of early breastfeeding cessation and may often reflect a mismatch between maternal expectations and normal infant feeding behavior rather than actual physiological insufficiency.

The exclusive breastfeeding rate of 29.1% falls below the WHO target of at least 50% (1). This rate is broadly consistent with estimates across Palestinian territories, where earlier data have documented exclusive breastfeeding rates ranging from approximately 25 to 40% (2, 4). Rates across the broader Arab and Eastern Mediterranean region are similarly below WHO targets, reflecting shared sociodemographic and healthcare-system-level barriers (3, 5).

The protective association between receipt of breastfeeding education and reduced formula use underscores the importance of structured educational interventions. Antenatal breastfeeding education may reinforce maternal intent, build confidence in milk supply sufficiency, and equip mothers with practical skills to manage common early breastfeeding challenges. The finding that antenatal breastfeeding education was independently protective is consistent with systematic reviews demonstrating efficacy of structured breastfeeding support (14, 15). However, the concurrent positive association of antenatal care attendance with formula use suggests that the quality and content of healthcare interactions are critical determinants of whether they reinforce or undermine breastfeeding (16).

The association between higher parity and formula use has been reported inconsistently in the literature. Some studies found multiparous mothers to have higher breastfeeding rates (17), while others documented patterns similar to our findings in contexts where domestic demands constrain exclusive breastfeeding (18). Early breastfeeding initiation has consistently been associated with improved outcomes (19, 20), and perceived low milk supply as a reason for formula introduction has been well documented across the Eastern Mediterranean (7, 20, 21).

Maternal employment and maternity leave policies are important structural determinants of infant feeding practices that warrant consideration in the Palestinian context. In our study, 25.9% of mothers cited maternal time constraints as the primary reason for formula introduction, and government and private employees had the highest formula use rates (86.7 and 84.0%, respectively) in bivariable analyses. These findings are consistent with evidence from the broader literature linking short or inadequate maternity leave with early breastfeeding cessation. In Palestine, maternity leave entitlements remain limited, and the absence of workplace lactation facilities represents a structural barrier that may compel employed mothers to transition to formula feeding prematurely (9, 15).

With respect to maternal nutrition, it is important to note that moderate undernutrition, which is common in food-insecure settings such as parts of the West Bank, does not generally reduce breast milk volume but may alter its micronutrient composition. Only severe malnutrition substantially affects milk production. In our study, maternal malnutrition (4.2% prevalence) was significantly associated with formula feeding in bivariable analysis (*p* = 0.044), though this association was attenuated in multivariable analysis (adjusted OR = 0.50, *p* = 0.425), likely reflecting small subgroup size. Healthcare providers should be cautious about recommending formula on the basis of perceived maternal undernutrition unless severe supply impairment is clinically confirmed, as this may inadvertently undermine breastfeeding in nutritionally vulnerable groups (20).

Regarding NICU admission and formula feeding, our data showed that 84.3% of neonates admitted to the NICU received formula, a proportion significantly higher than among non-admitted infants (68.8%, *p* = 0.008). It must be emphasized that the WHO recommends a mother’s own milk, or donor human milk, as the first-choice feeding option for hospitalized neonates, including those in intensive care settings. Formula feeding in the NICU context should be considered a medical decision of last resort rather than a default practice. Establishing or strengthening human milk banks and promoting kangaroo mother care within Palestinian NICUs could reduce unnecessary formula use in this vulnerable population (10).

Cesarean section was practiced in 26.7% of deliveries in our sample. Although cesarean delivery was not independently associated with formula feeding in multivariable analysis, the literature consistently documents that cesarean section delays the onset of breastfeeding, impairs early skin-to-skin contact, and is associated with shorter breastfeeding duration. Instrumental vaginal deliveries (forceps, vacuum) were not distinguished from spontaneous vaginal deliveries in our data collection instrument; this is acknowledged as a methodological limitation, as instrumental delivery may similarly interfere with early breastfeeding initiation. Future studies should capture this distinction explicitly (18).

Hospital maternity ward practices play a critical role in shaping early infant feeding behaviors and are an underexplored determinant in our study. Evidence from the Baby-Friendly Hospital Initiative (BFHI) demonstrates that practices such as immediate skin-to-skin contact, early initiation of breastfeeding within the first hour, rooming-in (mother and infant sharing the same room), avoidance of formula supplementation without medical indication, and structured breastfeeding support from trained staff significantly improve exclusive breastfeeding rates and duration (22). The extent to which the three participating PHCs and their affiliated hospitals adhere to BFHI principles was not assessed in this study; this represents an important gap. Auditing and strengthening these ward practices would likely complement community-based breastfeeding education efforts.

Our findings should also be interpreted within the broader framework of Infant and Young Child Feeding (IYCF), which encompasses not only breastfeeding and formula use but also complementary feeding practices and the overall nutritional adequacy of the diet in the first two years of life. Examining formula feeding in isolation provides an incomplete picture of infant nutrition; future research in Palestine should assess IYCF practices comprehensively (23). Furthermore, research among Syrian refugee populations in Lebanon and Jordan, communities that share many socioeconomic and health-system challenges with Palestinians, has highlighted the influential role of paternal attitudes, grandparental norms, and healthcare provider messaging in shaping breastfeeding decisions (24–26). These multi-level influences likely operate in the Palestinian context as well and should be explored in future qualitative and mixed-methods research.

This study has a cross-sectional design, so the results should be interpreted with this in mind. Several factors, such as attendance at antenatal care and breastfeeding education were significantly linked to formula feeding practices but care must be taken not to interpret the associations as causal links. The temporal order of exposure and outcome variables to the formula-feeding variables cannot be determined as these were measured concurrently. Hence, no conclusion could be drawn on whether the attendance of antenatal care or the breastfeeding education affected later feeding practices or whether there were other factors responsible for both. Moreover, the data were collected using consecutive sampling, which is a non-random sampling method, the prevalence in the study’s sample should be interpreted cautiously and should not be generalized to all mothers in the wider population. Furthermore, although the newly developed tool underwent validity assessment, its reliability was not formally evaluated; therefore, the internal consistency could not be established. Finally unmeasured variables like maternal beliefs and preferences, family and social support, past breastfeeding history, milk insufficiency perception, health care professionals’ advice, and socioeconomic status could have influenced the observed links. So, longitudinal studies are warranted in the future to determine if there are temporal relationships and to further understand the factors influencing formula-feeding practices.

### 4.1 Practical and policy implications

The high prevalence of mixed feeding and low rate of exclusive breastfeeding underscore an urgent need to strengthen breastfeeding promotion within Palestinian maternal health services. The independent protective association between breastfeeding education and reduced formula use provides a strong evidence base for scaling up antenatal breastfeeding counseling as a cost-effective policy lever.

The counterintuitive positive association between antenatal care and formula feeding suggests a critical need to audit the content of antenatal consultations. Baby-Friendly Hospital Initiative accreditation offers a structured framework for institutionalizing such quality improvements (8). Multiparous mothers with three or more children should be recognized as a high-risk group for formula introduction, warranting targeted postnatal breastfeeding support.

This study has several notable strengths. The sample size of 506 mothers provides adequate statistical power, and the breadth of variables collected enabled a comprehensive examination of factors associated with formula feeding. The use of multivariable logistic regression allowed simultaneous adjustment for multiple potential confounders. Multi-site recruitment across three geographically distinct governorates enhances representativeness. This study contributes to a population, Palestinian mothers, that remains substantially under-represented in the global infant feeding literature.

Several limitations should be acknowledged. The cross-sectional design precludes establishment of temporal relationships; reverse causality cannot be excluded. Feeding practices were self-reported, introducing the possibility of recall bias and social desirability bias. Selection bias may limit representativeness if participants were predominantly clinic attendees. Residual confounding from unmeasured variables, including paternal attitudes, household lactation support, and formula marketing exposure, may have influenced associations. Small cell sizes in certain chronic disease subgroups limited statistical power. The cross-sectional design also does not account for the dynamic and episodic nature of formula use; infants may have received formula during the first days of life, discontinued, and restarted later, and the current instrument cannot distinguish these patterns. Additionally, mode of delivery was dichotomised into vaginal versus cesarean section; instrumental vaginal deliveries (forceps, vacuum) were not captured separately and may influence early breastfeeding initiation. Maternal weight was collected without corresponding height measurement, precluding BMI calculation and limiting the interpretability of the weight variable as a proxy for nutritional status; future studies should include BMI. The back-translation of the questionnaire from Arabic into English, while enhancing semantic equivalence, may have introduced subtle linguistic or cultural nuances that were not fully captured, and the absence of formal psychometric validation of the instrument is an additional limitation affecting measurement precision. The generalizability of findings is further constrained by recruitment from PHC settings; mothers who do not attend such services may have different feeding practices.

Future research should prioritize prospective or longitudinal cohort studies following infants from birth to 24 months. Randomized or quasi-experimental trials evaluating structured antenatal breastfeeding education programs in Palestinian settings are needed. Qualitative research exploring maternal perceptions of milk insufficiency and the role of healthcare provider messaging would complement the quantitative findings. Multi-site studies spanning multiple Palestinian governorates would improve generalizability.

## 5 Conclusion

Mixed feeding was the predominant infant feeding pattern among Palestinian mothers in this study, with exclusive breastfeeding practiced by fewer than one-third of participants. Antenatal care attendance, multiparity, and the absence of breastfeeding education were independently associated with higher odds of formula use. These findings highlight antenatal breastfeeding education and targeted postnatal support for multiparous mothers as priority areas for intervention within Palestinian maternal health services. As a cross-sectional study, the findings reflect associations only and do not permit causal inference.

## Data Availability

The raw data supporting the conclusions of this article will be made available by the authors, without undue reservation.
