# Supplementary material for: Prevalence, determinants, and reasons for formula feeding among Palestinian mothers in the West Bank: a cross-sectional study
Source: Front Public Health. 2026 Sep 3;14:1908532. doi: 10.3389/fpubh.2026.1908532 (PMC13581779; doi:10.3389/fpubh.2026.1908532)
Supplement: Supplementary file 1 [file Table_1.DOCX]

**ممارسة التغذية بالحليب الصناعي والعوامل المرتبطة بها**

**بين الأمهات اللواتي لديهن أطفال بعمر من 0 إلى 24 شهراً في الضفة الغربية**

هذا الاستبيان جزء من دراسة بحثية تهدف إلى دراسة ممارسات التغذية بالحليب الصناعي والعوامل المرتبطة بها. مشاركتك طوعية وستبقى المعلومات سرية تماماً. يُرجى الإجابة على جميع الأسئلة بصدق.

**القسم الأول: المعلومات الديموغرافية للأم**

**1. عمر الأم:***

*حدد دائرة واحدة فقط.*

◯ أقل من 25 سنة

◯ من 25 إلى 34 سنة

◯ 35 سنة فأكثر

**2. عدد الأطفال:***

*حدد دائرة واحدة فقط.*

◯ طفل واحد

◯ طفلان

◯ 3 أطفال فأكثر

**3. الحالة الاجتماعية:***

*حدد دائرة واحدة فقط.*

◯ متزوجة

◯ مطلقة

◯ أرملة

**4. المستوى التعليمي:***

*حدد دائرة واحدة فقط.*

◯ أساسي / ثانوي (توجيهي)

◯ دبلوم جامعي

◯ بكالوريوس

◯ دراسات عليا (ماجستير / دكتوراه)

**5. الوضع الوظيفي:***

*حدد دائرة واحدة فقط.*

◯ ربة منزل

◯ طالبة

◯ موظفة حكومية

◯ موظفة في القطاع الخاص

◯ عمل حر

◯ مجال صحي

**القسم الثاني: الحالة الصحية للأم**

**6. هل تعانين من أمراض الغدة الدرقية؟***

*حدد دائرة واحدة فقط.*

◯ نعم

◯ لا

**7. هل تعانين من سوء التغذية / نقص في العناصر الغذائية؟***

*حدد دائرة واحدة فقط.*

◯ نعم

◯ لا

**8. هل عانيتِ من ارتفاع ضغط الدم خلال فترة الحمل؟***

*حدد دائرة واحدة فقط.*

◯ نعم

◯ لا

**9. هل تعانين من مرض السكري؟***

*حدد دائرة واحدة فقط.*

◯ نعم

◯ لا

**10. هل عانيتِ من سكري الحمل؟***

*حدد دائرة واحدة فقط.*

◯ نعم

◯ لا

**11. هل تعانين من مشاكل في القلب؟***

*حدد دائرة واحدة فقط.*

◯ نعم

◯ لا

**12. هل تعانين من أمراض أو مشاكل في الثدي (كتل، ورم، مشاكل في الحلمة، التهابات متكررة في الثدي)؟***

*حدد دائرة واحدة فقط.*

◯ نعم

◯ لا

**13. هل خضعتِ لعملية جراحية في الثدي سابقاً؟***

*حدد دائرة واحدة فقط.*

◯ نعم

◯ لا

**14. هل هناك تاريخ عائلي لأمراض وراثية؟***

*حدد دائرة واحدة فقط.*

◯ نعم

◯ لا

**15. هل عانيتِ من أي مشاكل طبية أخرى خلال الحمل؟***

*حدد دائرة واحدة فقط.*

◯ نعم

◯ لا

**القسم الثالث: معلومات إضافية عن الأم**

**16. طريقة الولادة:***

*حدد دائرة واحدة فقط.*

◯ ولادة طبيعية

◯ ولادة قيصرية

**17. الوزن الحالي للأم:***

*حدد دائرة واحدة فقط.*

◯ أقل من 60 كغم

◯ من 60 إلى 80 كغم

◯ أكثر من 80 كغم

**18. هل يعاني أحد أطفالك الآخرين من أمراض وراثية أو مزمنة؟***

*حدد دائرة واحدة فقط.*

◯ نعم

◯ لا

**القسم الرابع: معلومات حول الطفل الأخير**

*يتناول هذا القسم الحالة الصحية للطفل الحالي (آخر طفل للأم)، بالإضافة إلى حالته المتعلقة بالرضاعة والتغذية.*

**19. عمر الطفل:***

*حدد دائرة واحدة فقط.*

◯ دون عمر 3 أشهر

◯ من 3 أشهر إلى 6 أشهر

◯ من عمر 6 أشهر إلى 9 أشهر

◯ من 9 أشهر إلى 12 شهراً

◯ من 12 إلى 18 شهراً

◯ من 18 إلى 24 شهراً

**20. جنس الطفل:***

*حدد دائرة واحدة فقط.*

◯ ذكر

◯ أنثى

**21. العمر الجسدي عند الولادة (عمر الحمل):***

*حدد دائرة واحدة فقط.*

◯ أقل من 36 أسبوعاً (خداج / مبتسر)

◯ من 36 إلى 40 أسبوعاً (طبيعي)

◯ أكثر من 40 أسبوعاً (ما بعد الطبيعي)

**22. هل حدثت أي مضاعفات أثناء الولادة؟***

*حدد دائرة واحدة فقط.*

◯ نعم

◯ لا

**23. متى بدأتِ الرضاعة الطبيعية؟***

*حدد دائرة واحدة فقط.*

◯ خلال الساعة الأولى من الولادة

◯ خلال أول يوم

◯ من 1 إلى 3 أيام

◯ بعد أكثر من 3 أيام

**24. هل واجهتِ أي مضاعفات صحية بعد الولادة (مثل: نزيف، ارتفاع حرارة الجسم، انسداد أوعية دموية، عدوى)؟***

*حدد دائرة واحدة فقط.*

◯ نعم

◯ لا

**25. هل دخل الطفل إلى الحضانة / العناية المركزة بعد الولادة؟***

*حدد دائرة واحدة فقط.*

◯ نعم

◯ لا

**26. وزن الطفل عند الولادة:***

*حدد دائرة واحدة فقط.*

◯ دون 2500 غم (2.5 كغم) — وزن منخفض

◯ من 2500 إلى 4000 غم (2.5 – 4 كغم) — طبيعي

◯ أكثر من 4000 غم (أكثر من 4 كغم) — كبير الحجم

**27. هل تلقّى الطفل رضاعة طبيعية؟***

*حدد دائرة واحدة فقط.*

◯ نعم

◯ لا

**القسم الخامس: ممارسات التغذية بالحليب الصناعي**

**28. متى تم إدخال الحليب الصناعي؟***

*حدد دائرة واحدة فقط.*

◯ خلال الساعة الأولى من الولادة

◯ خلال أول يوم

◯ من 1 إلى 3 أيام

◯ من 3 أيام إلى شهر واحد

◯ من 1 إلى 3 أشهر

◯ من 3 إلى 6 أشهر

◯ من 6 إلى 12 شهراً

**29. نوع التغذية الحالية للطفل:***

*حدد دائرة واحدة فقط.*

◯ رضاعة طبيعية حصرية

◯ حليب صناعي حصري

◯ تغذية مختلطة (طبيعية وصناعية)

**30. إذا كانت هناك رضاعة طبيعية، كم استمرت الرضاعة الطبيعية الحصرية؟***

*حدد دائرة واحدة فقط.*

◯ أقل من شهر

◯ من 1 إلى 3 أشهر

◯ من 3 إلى 6 أشهر

◯ أكثر من 6 أشهر

◯ لا تنطبق (لم تكن هناك رضاعة طبيعية حصرية)

**31. ما هو سبب التوجه إلى الرضاعة الصناعية؟**

*حدد دائرة واحدة فقط.*

◯ نقص في حليب الثدي (كمية الحليب قليلة)

◯ عدم تقبل الطفل للحليب الطبيعي

◯ نصيحة من الأقارب

◯ نصيحة من مراكز الرعاية / صيدليات

◯ من أجل المحافظة على شكل الثدي

◯ الانشغال في العمل أو الدراسة

◯ عن طريق دعايات إعلانية

**أسباب أخرى، يرجى التوضيح:**

**القسم السادس: الرعاية السابقة للولادة والتثقيف الصحي**

**32. هل تابعتِ طبيب الرعاية خلال فترة الحمل؟***

*حدد دائرة واحدة فقط.*

◯ نعم

◯ لا

**33. كم عدد الزيارات للطبيب خلال فترة الحمل؟***

*حدد دائرة واحدة فقط.*

◯ أقل من 3 مرات

◯ 3 مرات أو أكثر

**34. هل تلقّيتِ توعية بالرضاعة الطبيعية خلال فترة الحمل أو بعدها؟***

*حدد دائرة واحدة فقط.*

◯ نعم

◯ لا

**شكراً جزيلاً على مشاركتك القيّمة في هذه الدراسة**
