# Supplementary material for: Prevalence, determinants, and reasons for formula feeding among Palestinian mothers in the West Bank: a cross-sectional study
Source: Front Public Health. 2026 Sep 3;14:1908532. doi: 10.3389/fpubh.2026.1908532 (PMC13581779; doi:10.3389/fpubh.2026.1908532)
Supplement: Supplementary file 2 [file Table_2.DOCX]

**Formula Feeding Practices and Associated Factors**

**Among Mothers with Infants Aged 0–24 Months in the West Bank**

This questionnaire is part of a research study examining formula feeding practices and their associated factors. Your participation is voluntary and all information will be kept strictly confidential. Please answer all questions honestly.

**Section 1: Maternal Sociodemographic Information**

**1. Maternal age:***

*Select one circle only.*

◯ Less than 25 years

◯ 25 to 34 years

◯ 35 years or older

**2. Number of children (parity):***

*Select one circle only.*

◯ One child

◯ Two children

◯ Three children or more

**3. Marital status:***

*Select one circle only.*

◯ Married

◯ Divorced

◯ Widowed

**4. Educational level:***

*Select one circle only.*

◯ Basic / Secondary (Tawjihi)

◯ University diploma

◯ Bachelor's degree

◯ Postgraduate (Master's / Doctorate)

**5. Employment status:***

*Select one circle only.*

◯ Housewife

◯ Student

◯ Government employee

◯ Private sector employee

◯ Freelance

◯ Healthcare field

**Section 2: Maternal Health Status**

**6. Do you have thyroid disease?***

*Select one circle only.*

◯ Yes

◯ No

**7. Do you have malnutrition or nutritional deficiency?***

*Select one circle only.*

◯ Yes

◯ No

**8. Did you experience high blood pressure (hypertension) during pregnancy?***

*Select one circle only.*

◯ Yes

◯ No

**9. Do you have diabetes mellitus?***

*Select one circle only.*

◯ Yes

◯ No

**10. Did you experience gestational diabetes?***

*Select one circle only.*

◯ Yes

◯ No

**11. Do you have any heart problems?***

*Select one circle only.*

◯ Yes

◯ No

**12. Do you have any breast diseases or problems (lumps, tumours, nipple problems, recurrent breast infections)?***

*Select one circle only.*

◯ Yes

◯ No

**13. Have you had any previous breast surgery?***

*Select one circle only.*

◯ Yes

◯ No

**14. Is there a family history of genetic disorders?***

*Select one circle only.*

◯ Yes

◯ No

**15. Did you have any other medical problems during pregnancy?***

*Select one circle only.*

◯ Yes

◯ No

**Section 3: Additional Maternal Information**

**16. Mode of delivery:***

*Select one circle only.*

◯ Normal vaginal delivery

◯ Caesarean section

**17. Current maternal weight:***

*Select one circle only.*

◯ Less than 60 kg

◯ 60 to 80 kg

◯ More than 80 kg

**18. Do any of your other children suffer from a genetic or chronic disease?***

*Select one circle only.*

◯ Yes

◯ No

**Section 4: Information About the Most Recent Child**

*This section covers the health status of the current child (the mother's most recent child), including breastfeeding and feeding status.*

**19. Child's age:***

*Select one circle only.*

◯ Under 3 months

◯ 3 to 6 months

◯ 6 to 9 months

◯ 9 to 12 months

◯ 12 to 18 months

◯ 18 to 24 months

**20. Child's sex:***

*Select one circle only.*

◯ Male

◯ Female

**21. Gestational age at delivery:***

*Select one circle only.*

◯ Less than 36 weeks (preterm)

◯ 36 to 40 weeks (term)

◯ More than 40 weeks (post-term)

**22. Were there any complications during delivery?***

*Select one circle only.*

◯ Yes

◯ No

**23. When was breastfeeding initiated?***

*Select one circle only.*

◯ Within the first hour after birth

◯ Within the first day

◯ 1 to 3 days after birth

◯ More than 3 days after birth

**24. Did you experience any health complications after delivery (e.g., bleeding, fever, blood clot, infection)?***

*Select one circle only.*

◯ Yes

◯ No

**25. Was the infant admitted to the neonatal intensive care unit (NICU) after birth?***

*Select one circle only.*

◯ Yes

◯ No

**26. Birth weight:***

*Select one circle only.*

◯ Less than 2,500 g (< 2.5 kg) — Low birth weight

◯ 2,500 to 4,000 g (2.5–4 kg) — Normal

◯ More than 4,000 g (> 4 kg) — Macrosomia

**27. Did the child receive any breastfeeding?***

*Select one circle only.*

◯ Yes

◯ No

**Section 5: Formula Feeding Practices**

**28. When was infant formula first introduced?***

*Select one circle only.*

◯ Within the first hour of birth

◯ Within the first day

◯ 1 to 3 days

◯ 3 days to 1 month

◯ 1 to 3 months

◯ 3 to 6 months

◯ 6 to 12 months

**29. Current feeding type:***

*Select one circle only.*

◯ Exclusive breastfeeding

◯ Exclusive formula feeding

◯ Mixed feeding (breastfeeding and formula)

**30. If breastfed, how long was exclusive breastfeeding maintained?***

*Select one circle only.*

◯ Less than 1 month

◯ 1 to 3 months

◯ 3 to 6 months

◯ More than 6 months

◯ Not applicable (no exclusive breastfeeding)

**31. What was the reason for switching to formula feeding?**

*Select one circle only.*

◯ Insufficient breast milk (low milk supply)

◯ Infant's refusal of breast milk

◯ Advice from relatives

◯ Advice from healthcare centres / pharmacies

◯ To maintain breast shape

◯ Work or study commitments

◯ Exposure to advertising

**Other reasons, please specify:**

**Section 6: Antenatal Care and Health Education**

**32. Did you attend antenatal care during pregnancy?***

*Select one circle only.*

◯ Yes

◯ No

**33. How many antenatal visits did you attend during pregnancy?***

*Select one circle only.*

◯ Fewer than 3 visits

◯ 3 visits or more

**34. Did you receive breastfeeding education during or after pregnancy?***

*Select one circle only.*

◯ Yes

◯ No

**Thank you sincerely for your valuable participation in this study.**
