# Supplementary material for: Prevalence, determinants, and reasons for formula feeding among Palestinian mothers in the West Bank: a cross-sectional study
Source: Front Public Health. 2026 Sep 3;14:1908532. doi: 10.3389/fpubh.2026.1908532 (PMC13581779; doi:10.3389/fpubh.2026.1908532)
Supplement: Supplementary file 3 [file Table_3.DOCX]

| **Variable** | **Operational Definition** |
| --- | --- |
| **Formula feeding (primary outcome)** | Introduction of any commercial infant formula milk during the infant's first 12 months of life, regardless of whether it was used as a supplement to or replacement for breastfeeding. Categorized as **Yes** or **No**. |
| **Type of milk feeding** | Infant feeding pattern before 12 months classified into three mutually exclusive categories: **exclusive breastfeeding**, **exclusive formula feeding**, or **mixed feeding (breastfeeding and formula feeding)**. |
| **Maternal age group** | Mother's age categorized as **<25 years**, **25–34 years**, or **≥35 years**. |
| **Marital status** | Self-reported marital status categorized as **married**, **divorced**, or **widowed**. |
| **Educational level** | Highest educational qualification attained, categorized as **basic education**, **secondary school**, **diploma**, **bachelor's degree**, or **master's/PhD**. |
| **Employment status** | Mother's current occupation categorized as **housewife**, **student**, **government employee**, **private employee**, **freelancer**, or **health-related profession**. |
| **Parity (number of children)** | Total number of living children categorized as **1 (primiparous)**, **2**, or **≥3 children**. |
| **Family history of genetic disorders** | Self-reported presence of a family history of genetic disorders, categorized as **Yes** or **No**. |
| **Maternal weight** | Self-reported maternal body weight categorized as **<60 kg**, **60–80 kg**, or **>80 kg**. |
| **Hypertension** | Self-reported physician diagnosis of hypertension (Yes/No). |
| **Diabetes mellitus** | Self-reported physician diagnosis of diabetes mellitus (Yes/No). |
| **Heart disease** | Self-reported physician diagnosis of heart disease (Yes/No). |
| **Breast disorder** | Self-reported history of breast disease or disorder (Yes/No). |
| **Thyroid disease** | Self-reported physician diagnosis of thyroid disease (Yes/No). |
| **Malnutrition** | Self-reported maternal malnutrition (Yes/No). |
| **Prior breast surgery** | Self-reported history of breast surgery (Yes/No). |
| **Infant age** | Infant's age at the time of the survey categorized as **0–3**, **3–6**, **6–9**, **9–12**, **12–18**, or **18–24 months**. |
| **Infant sex** | Infant sex categorized as **male** or **female**. |
| **Timing of breastfeeding initiation** | Time elapsed from birth until first breastfeeding categorized as **within 1 hour**, **within 1 day**, **1–3 days**, or **>3 days**. |
| **Timing of formula introduction** | Infant age at first exposure to formula categorized as **within 1 hour**, **within 1 day**, **1–3 days**, **3 days–1 month**, **1–3 months**, **3–6 months**, or **6–12 months**. |
| **Mode of delivery** | Delivery classified as **vaginal delivery** or **caesarean section**. |
| **Delivery complications** | Presence of any delivery-related complication, categorized as **Yes** or **No**. |
| **NICU admission** | Admission of the newborn to a neonatal intensive care unit after birth (Yes/No). |
| **Birth weight** | Birth weight categorized as **<2,500 g (low birth weight)**, **2,500–4,000 g (normal)**, or **>4,000 g (macrosomia)**. |
| **Gestational age at delivery** | Gestational age categorized as **<36 weeks (preterm)**, **36–40 weeks (term)**, or **>40 weeks (post-term)**. |
| **Antenatal care attendance** | Whether the mother received antenatal care during pregnancy (Yes/No). |
| **Number of antenatal visits** | Total antenatal care visits categorized as **<3 visits** or **≥3 visits**. |
| **Pregnancy complications** | Presence of any medical problem during pregnancy (Yes/No). |
| **Hypertensive disorders of pregnancy** | Self-reported hypertensive disorder during pregnancy (Yes/No). |
| **Gestational diabetes mellitus** | Self-reported diagnosis of gestational diabetes during pregnancy (Yes/No). |
| **Breastfeeding education** | Receipt of antenatal breastfeeding education or counselling, categorized as **Yes** or **No**. |
| **Any breastfeeding** | Mother reported breastfeeding her infant at any time after birth (Yes/No). |
| **Any formula feeding** | Mother reported introducing formula milk at any time during infancy (Yes/No). |
| **Primary reason for formula introduction** | Mother's main self-reported reason for introducing formula, categorized as **perceived low milk supply**, **maternal time constraints**, **advice from healthcare providers**, **other medical conditions**, **infant refusal of the breast**, or **advice from relatives**. |
